# Pesticide exposure and lymphohaematopoietic cancers: a case-control study in an agricultural region (Larissa, Thessaly, Greece)

**DOI:** 10.1186/1471-2458-11-5

**Published:** 2011-01-04

**Authors:** Maria Kokouva, Nikolaos Bitsolas, Georgios M Hadjigeorgiou, George Rachiotis, Nikolaos Papadoulis, Christos Hadjichristodoulou

**Affiliations:** 1 Department of Hygiene and Epidemiology, Medical Faculty, University of Thessaly, Larissa, Greece; 2 Department of Neurology, Medical Faculty, University of Thessaly, Larissa, Greece; 3 Department of Haematology and Blood Donors, General Hospital of Larissa, Larissa, Greece

## Abstract

**Background:**

The causality of lymphohaematopoietic cancers (LHC) is multifactorial and studies investigating the association between chemical exposure and LHC have produced variable results. The aim of this study was to investigate the relationships between exposure to pesticides and LHC in an agricultural region of Greece.

**Methods:**

A structured questionnaire was employed in a hospital-based case control study to gather information on demographics, occupation, exposure to pesticides, agricultural practices, family and medical history and smoking. To control for confounders, backward conditional and multinomial logistic regression analyses were used. To assess the dose-response relationship between exposure and disease, the chi-square test for trend was used.

**Results:**

Three hundred and fifty-four (354) histologically confirmed LHC cases diagnosed from 2004 to 2006 and 455 sex- and age-matched controls were included in the study. Pesticide exposure was associated with total LHC cases (OR 1.46, 95% CI 1.05-2.04), myelodysplastic syndrome (MDS) (OR 1.87, 95% CI 1.00-3.51) and leukaemia (OR 2.14, 95% CI 1.09-4.20). A dose-response pattern was observed for total LHC cases (P = 0.004), MDS (P = 0.024) and leukaemia (P = 0.002). Pesticide exposure was independently associated with total LHC cases (OR 1.41, 95% CI 1.00 - 2.00) and leukaemia (OR 2.05, 95% CI 1.02-4.12) after controlling for age, smoking and family history (cancers, LHC and immunological disorders). Smoking during application of pesticides was strongly associated with total LHC cases (OR 3.29, 95% CI 1.81-5.98), MDS (OR 3.67, 95% CI 1.18-12.11), leukaemia (OR 10.15, 95% CI 2.15-65.69) and lymphoma (OR 2.72, 95% CI 1.02-8.00). This association was even stronger for total LHC cases (OR 18.18, 95% CI 2.38-381.17) when eating simultaneously with pesticide application.

**Conclusions:**

Lymphohaematopoietic cancers were associated with pesticide exposure after controlling for confounders. Smoking and eating during pesticide application were identified as modifying factors increasing the risk for LHC. The poor pesticide work practices identified during this study underline the need for educational campaigns for farmers.

## Background

The causality of the various types of lymphohaematopoietic cancers (LHC) is multifactorial and includes physical, chemical and biological agents, for example radiation, solvents, viruses etc. In addition, there are a number of agents that are suspected causes of LHC [1,2].

Several studies have suggested that occupational exposure is associated with LHC. In particular, farming has been associated with non-Hodgkin lymphoma (NHL), chronic myeloid leukaemia, multiple myeloma, and Hodgkin's disease (HD) [3-7]. Animal breeding, including poultry rearing, has been associated with almost all types of lymphatic cancers: low grade NHL, chronic lymphoid leukaemia, and acute myeloid leukaemia [8-10]. Chemical and woodworking industries were found to increase the incidence of HD whereas solvent exposure, especially chlorinated hydrocarbons, was found to be associated with malignant lymphoma [11]. Specific chromosomal translocations of NHL in farmers, such as 14q32 among fumigant appliers and 18q21 among herbicide appliers were observed in some studies [12-14]. Finally, in several studies various chemicals/pesticides were found to be associated with LHC. Phenoxy herbicides (especially 2,4 D [2,4-dichlorophenoxy acetic acid]), chlordane termiticides, mothballs, dicamba-containing herbicides, mecoprop, aldrin and fungicides were related to NHL [15,16]. Carbamates and phosphates were associated with chronic lymphoid leukaemia and NHL [17]. Non-arsenic pesticides were related to lymphoma [18]. Triazol fungicides and urea herbicides have been associated with HD and insecticides, fungicides and herbicides with multiple myeloma [19].

Pesticides, which are widely used in agriculture, comprise a wide variety of chemicals mainly used to control damage to plants from insects (insecticides), mold (fungicides) and weeds (herbicides).

The aim of this study was to investigate the relationships between exposure to pesticide and LHC in the region of Thessaly (central Greece), which has a substantial agricultural labour force. The study investigated application procedures as well as the use of personal protective equipment (PPE) in farming and quantified exposure using detailed questionnaires administered to patients and controls from two hospitals in the Thessaly region.

## Methods

### Study setting

A case control study of LHC was designed and conducted in two regional hospitals of Larissa (Thessaly, Greece) from October 2003 to October 2006. Thessaly is one of the thirteen regions in the central eastern part of continental Greece, comprising the prefectures of Larissa, Karditsa, Trikala and Magnesia. The total area is 14,036 square kilometers (Km^2^), which represents 10.6% of the area of the entire country. According to the last census conducted in 2001 by the General Secretariat of the National Statistical Service of Greece (ESYE), the population of the region was approximately 740,000 inhabitants, which represents 7% of the total population of the country [20]. The population break down is 44% urban, 16% semi-urban and 40% rural. Thessaly ranks second among the agricultural producing regions of Greece (after Central Macedonia) and contributes 14.2% of the total primary sector produce of the country.

### Cases

Five hundred and ninety-seven (597) histologically confirmed cases of myeloproliferative disorder (MPD), myelodysplastic syndrome (MDS), leukaemia, lymphoma and plasmacell disease (PcD) were collected from the medical records of the two hospitals within a two-years period. The FAB (French American British) and REAL (Revised European-American Lymphoma) classifications were used, since both were used by the attending physicians during the study period.

During the data collection period, 35 cases died before entering the study. There were also new cases enrolled (16), and a number (102) of cases could not be identified due to data missing from the medical records (incorrect address or phone number etc). Out of the 476 available prevalent cases, 354 agreed to participate in the study and completed the questionnaire (response rate 74.36%). Moreover, 325 cases provided blood samples for further studies.

### Controls

The control group comprised subjects from the same hospitals who were admitted for acute conditions such as orthopaedic, eye, ear, nose, throat or surgical diagnoses and acute cardiac conditions. Some subjects were also recruited from the internal medicine and blood donor departments. All of them were self-declared cancer-free patients. Cases were divided into groups with age ranges of 5 years (e.g. 60-64, 65-69 etc) and were matched according to age and sex.

Almost all controls who were asked to participate completed the questionnaire (response rate 93.2%), and 408 out of 455 provided blood samples for further studies.

### Questionnaire

To ascertain information from both cases and controls a structured questionnaire was developed and pre-tested (see Additional file 1).

The questionnaire included six different sections: 1) demographics, 2) residence, 3) occupation, 4) exposure and agricultural practices, 5) family history and 6) habits and medical history. The demographic section included the hospital where treatment was obtained, level of education and type of insurance. The residence section included full address, chronological residential changes with duration of stay and residential level (urban, suburban or rural). The assignment of the residential level and the coding of the residence were performed using lists from the Ministry of Internal Affairs of Greece. In the occupational section, detailed information on previous employment and other activities were reported.

Subjects who reported at least one year of work in farming were asked to complete the part of the questionnaire on pesticide exposure. The information collected included type of crop, duration of farming, surface area of the farm, characteristics of the adjacent farm and crop infestations. The participants were asked to provide commercial names of pesticide products, number of applications per year, total years of use and type of application (spraying, during planting and usage of treated seeds). To provide help to the participants in recalling the pesticides used, a list of the most common brand names was provided. Moreover, questions were included regarding adherence to application guidelines and familiarity with caution signs/first aid help. The type and purpose of the use of PPE (e.g. uniforms, masks, boots etc) the use of equipment machinery, the frequency of consulting experts, aspects of product handling (e.g. location of storage) and the existence of an integrated farm system were also investigated. In addition, information on certain habits such as changing clothes before entering the house, smoking, eating and accidents (e.g. accidental poisoning), was also collected. Finally, details of work with animals and pet ownership were considered.

In the family history section, all types of cancers, LHC and immunological disorders as well as the family proximity (first, second and third degree relatives) were recorded. Among the immunological disorders rheumatoid arthritis, Sjögren's syndrome, systemic lupus erythematosus, inflammatory bowel diseases, sarcoidosis, psoriasis and atopic conditions were included.

Categories for smoking habit and alcohol consumption according to the Centers for Diseases Control and Prevention (CDC) 2002 classifications were adopted [21]. Information regarding the subject's medical history included other cancers, cardiovascular disorders, respiratory diseases, immunological disorders, inflammatory diseases, endocrine disorders, diabetes mellitus and other metabolic disorders.

The questionnaire was pilot tested among both cases and controls aged 27 to 73 years and modifications were made according to the results obtained. The time needed to fill the questionnaire was reasonable with the only delay at the section regarding the specific pesticides used. The interviews were conducted by a trained occupational physician and the date of diagnosis was used as a reference date for both cases and controls.

Due to changes in pesticide exposure over time, an expert (phytopathologist) evaluated each questionnaire and the data collected regarding pesticide exposure and handling practices, as well as commercial names of pesticides and types of application. All active ingredients were grouped according to the target pest into three major categories: 1) insecticides (organophosphates, carbamates, organochlorines, pyrethroids, avermectins etc), 2) fungicides (inorganic composition e.g. sulfur, disulfide carbamates, benzo-imidazolic composition etc) and 3) herbicides (phenoxy acids, urea products, triazines, bipyridine, nitroanilines etc). According to the chemical composition, the active ingredients were grouped into another three categories: 1) carbamates (carbaryl, propoxur, mancozeb, maneb, thiram, ziram etc), 2) organophosphates (monocrotophos, dimethoate, methamidophos, parathion, chlorpyrifos, methidathion, phosmet etc) and 3) organochlorines (dicofol, dieldrin, heptachlor, endrin, endosulfan etc) [22].

A new variable was developed for total pesticide exposure (residential, environmental and occupational) for all subjects, in order to classify them in the "Low/No", "Medium" or "High" category. Participants who had never worked in the agricultural sector as their main or secondary occupation and declared urban or suburban residences were classified in the "Low/No" exposure category. For the "Medium" and "High" exposure categories the following equation for total pesticide exposure was developed: number of treatments per year multiplied by total lifetime years of use multiplied by area cultivated (Km^2^). To classify participants in the "Medium" or "High" exposure category, a cutoff point of 60,000 y·Km^2 ^was set, which is approximately the mean value for total pesticide exposure (59,900 y·Km^2^). Moreover for participants close to the cut-off value, the following exposure modifying factors were taken into consideration before final categorization in the "Medium" or "High" category: 1) application of pesticides by the person, which possibly enhanced exposure, was considered to be the major exposure modifying factor and, for this reason, the value obtained from the above equation was multiplied by the factor 1.3 2) the type of equipment used during application, which possibly reduced exposure, was considered the second most important exposure modifying factor and, for this reason, the value obtained from the above equation was multiplied by 0.8 3) the use of PPE was considered of minor importance in our assessment for the classification of participants into the "Medium" or "High" exposure category given the improper use of PPE and, in this case, the value obtained from the above equation was multiplied by 0.9.

In order to avoid reporting exposure that occurred after LHC diagnosis and to allow a minimum latency period, the period preceding one year before the reference date for both cases and controls was used for recorded exposure.

### Statistical analysis

Standard statistical procedures were carried out using the epidemiological software Epi-info (version 3.4.3.) and the Statistical Package for Social Sciences (SPSS) (version 15.0). Data were validated for completeness before statistical analysis was performed. Descriptive analysis was conducted for each variable including frequencies, ranges, means ± SD, median values and interquartile ranges (IQR) for both cases and controls. The chi-square or Fisher exact test was used to analyze qualitative data, whereas the student *t*-test or Mann-Whitney test was used for quantitative data. Total pesticide exposure and smoking habit as well as smoking and eating during farming were made categorical (yes/no) variables. Agricultural occupations were grouped together (farmer, pesticide applier, seasonal farm worker, animal breeder) and compared to the group of other occupations (e.g. teacher, trader, public servant, unemployed etc.) Backward conditional logistic regression analysis was used to control for confounders and to identify independent risk factors. Exposure to pesticides was an independent categorical variable. Each histological group and the group comprising all LHC cases were analyzed separately. To assess the dose-response relationship between exposure and disease, the chi square test for trend was used. Moreover, age, sex, smoking and family history (all types of cancer, LHC and immunological disorders) were included as confounders. Afterwards, a multinomial logistic regression (polytomous regression) model was used to evaluate whether the risk factors differ across the histological groups and the total LHC group [23]. In this model LHC was the dependent variable, and pesticide exposure was the independent categorical variable. Smoking during application, age and sex were confounders. All control subjects were included in the statistical analysis of each histological group. The analysis was conducted separately for the five histological groups (Group 1: MPD, Group 2: MDS, Group 3: leukaemia, Group 4: lymphoma and Group 5: PcD) and for all the LCH cases. Differences were considered statistically significant when the two-sided p value was ≤ 0.05.

### Ethics

The study was approved by the University of Thessaly Scientific Committee which is responsible for ethics. The Institutional Scientific Committee of both hospitals approved access to patients' files and allowed interviews. The participants provided informed verbal consent and no monetary incentives for participation were offered.

## Results

Overall, 354 cases (69 MPD, 78 MDS, 74 leukaemia, 75 lymphoma, and 58 PcD) and 455 controls (81 orthopaedics, 84 otolaryngology, 64 ophthalmology, 93 surgery, 42 cardiology, 59 internal medicine and 32 blood donors) were collected. In Table 1 the characteristics of the study population, including sex, agricultural occupation, pesticide exposure and smoking habit, are presented. The median age of both cases and controls was 70 years (IQR 62-76).

**Table 1:** Description of the study population.

		Cases		Controls	
Sex	Male	188	53.10%	238	52.30%
	Female	166	46.90%	217	47.70%
Agricultural occupation	Yes	185	52.25%	212	46.60%
	No	169	47.75%	243	53.40%
Pesticide exposure	Yes	282	79.66%	331	72.74%
	Low/No	72	20.34%	124	27.26%
Smoking	Yes	148	41.80%	205	45.05%
	No	206	58.20%	250	54.95%
Total		354		455	

As shown in Table 2 univariate analysis revealed statistically significant associations between exposure to pesticides and all cases of LHC (OR 1.46, 95% CI 1.05 - 2.04), MDS (OR 1.87, 95% CI 1.00-3.51) and leukaemia (OR 2.14, 95% CI 1.09 - 4.20). A dose-response effect was observed for almost all histological groups and was statistically significant for all cases of LHC (P = 0.004), MDS (P = 0.024) and leukaemia (P = 0.002).

**Table 2:** Univariate analysis of overall previous pesticide exposure among LHC cases and controls.

Exposure Status	Controls	LHC Cases	MPD	MDS	Leukaemia	Lymphoma	PcD
Low/No (Ref)	124 (27.2%)	72 (20.3%)	18 (26.1%)	13 (16.7%)	11 (14.9%)	20 (26.7%)	10 (17.2%)
Medium	140 (30.8%)	99 (28.0%)	15 (21.7%)	23 (29.5%)	18 (24.2%)	24 (32.0%)	19 (32.8%)
High	191 (42.0%)	183 (51.7%)	36 (52.2%)	42 (53.8%)	45 (60.9%)	31 (41.3%)	29 (50.0%)

OR Medium vs Low/No (95% CI)	Ref	1.22 (0.81-1.83)	0.74 (0.35-1.73)	1.57 (0.72-3.43)	1.45 (0.62-3.43)	1.06 (0.54-2.12)	1.68 (0.71-4.05)
OR High vs Low/No (95% CI)	Ref	1.65 (1.14-2.39)	1.37 (0.71-2.68)	2.10 (1.04-4.30)	2.66 (1.27-5.68)	1.01 (0.62-2.23)	1.88 (0.84-4.30)

OR Exposed vs Non exposed (Medium and High vs Low/No) (95% CI)	Ref	1.46* (1.05-2.04)	1.06 (0.59-1.88)	1.87* (1.00-3.51)	2.14* (1.09-4.20)	1.03 (0.59-1.79)	1.79 (0.88-3.66)

* P value ≤0.05

Leukaemia (OR 1.68, 95% CI 1.02-2.76) and MDS (OR 1.73, 95% CI 1.06-2.83) were associated with agricultural occupation when compared to other occupations as described in Table 3.

**Table 3:** Logistic regression analysis of occupational status and LHC cases and histological groups

	Controls	LHC Cases	MPD	MDS	Leukaemia	Lymphoma	PcD
**Agricultural occupation**	212	185	36	47	44	31	27
**Other occupations**	243	169	33	31	30	44	31
**OR**	Ref	1.25	1.25	1.73*	1.68*	0.80	0.99
**95% CI**		0.95-1.65	0.75-2.07	1.06-2.83	1.02-2.76	0.49-1.32	0.57-1.72

*Pvalue ≤0.05

There were no statistically significant differences between the three pesticide categories according to target pest (insecticides, fungicides and herbicides) and the three chemical categories (carbamates, organophosphates and organochlorines) with respect to the histological groups or the total LHC cases (data not shown).

Two notable findings regarding the type of application were found (Table 4). The first was the positive association of seed treatment with MDS (OR 5.06, 95% CI 1.53-16.75) and the second was the negative/protective association of spraying with MPD (OR 0.34, 95% CI 0.13-0.95) and MDS (OR 0.33, 95% CI 0.13-0.81) for the participants who manage to recall in details the pesticide products used and type of application for each.

**Table 4:** Type of pesticide application for each histological group compared to healthy controls.

	Spraying			During planting			Seed investment		
	
	n	OR	95% CI	n	OR	95% CI	n	OR	95% CI
**LHC cases**	123	0.40*	0.22-0.74	20	1.87	0.87-4.07	18	3.01*	1.19-7.77
**MPD**	22	0.34 *	0.13-0.95	5	2.64	0.76-8.8	3	2.65	0.52-12
**MDS**	29	0.33 *	0.13-0.81	4	1.47	0.38-5.16	7	5.06 *	1.53-16.75
**Leukaemia**	33	0.46	0.18-1.10	4	1.39	0.36-4.88	5	3.23	0.86-11.71
**Lymphoma**	25	0.52	0.18-1.58	5	2.54	0.73-8.43	1	0.65	0.13-5.35
**PcD**	14	0.75	0.22-2.80	2	1.02	0.00-5.12	2	1.84	0-10.16
**Controls**	177	Ref		14	Ref		8	Ref	

*Pvalue ≤0.05

Regarding the various agricultural practices (Table 5), failure to change clothes after work was statistically significantly associated with LHC (OR 1.51, 95% CI 1.00-2.29). Furthermore, the lack of proper ventilation of the pesticide storage location was identified as a possible risk factor related to total LHC cases (OR 3.60, 95% CI 1.77-7.31).

**Table 5:** Agricultural practices of total LHC cases compared to healthy controls.

Agricultural practice	OR	95% CI
Compliance with product labels	0.85	0.56-1.28
Familiarity with caution signs	1.05	0.66-1.65
Familiarity with first aid help	1.22	0.34-4.26
Vicinity of first aid station	0.75	0.47-1.19
Self application of pesticides	1.14	0.82-1.60
Use of PPE	1.10	0.74-1.63
Use of PPE during preparation	0.72	0.44-1.16
Use of PPE during application	0.86	0.53-1.38
Use of PPE after application	0.62	0.27-1.42
Use of PPE during cleaning of equipment	1.52	0.52-4.42
Use of equipment	1.11	0.80-1.55
Not changing clothes after work	1.51*	1.00-2.29
Accidental poisoning	0.89	0.51-1.86
Expert's advice	0.86	0.56-1.28
Buying excessive quantity of product	1.16	0.79-1.72
Appropriate handling of eluants	5.66	0.33-96.89
Separate storage	0.35	0.92-1.19
No proper ventilation	3.60*	1.77-7.31
Animal breeding	1.23	0.92-1.62

*P value ≤0.05

No association was found between the various types of crops cultivated (cereal crops, combinable crops, tree fruits, legume vegetables, and decorative plants) and total LHC cases.

The most consistent results involved the smoking habit. As shown in Table 6 smoking (current and former smokers) during pesticide application was significantly associated with cases of LHC (OR 3.29, 95% CI 1.81-5.98), MDS (OR 3.67, 95% CI 1.18-12.11), leukaemia (OR 10.15, 95% CI 2.15-65.69) and lymphoma (OR 2.72, 95% CI 1.02-8.00).

**Table 6:** Logistic regression analysis of smoking during application of pesticides, LHC cases and histological groups.

	Smoking during application	Non-smoking during application	OR	95% CI
LHC Cases	75 (55.6%)	22 (27.5%)	3.29*	1.81-5.98
MPD	10 (14.28%)	5 (7.93%)	1.93	0.56-6.98
MDS	19 (24.05%)	5 (7.93%)	3.67*	1.18-12.11
Leukaemia	21 (25.92%)	2 (3.33%)	10.15*	2.15-65.69
Lymphoma	17 (20.07%)	6 (9.37%)	2.72*	1.02-8.00
PcD	8 (11.76%)	4 (6.45%)	1.93	0.49-8.14
				
Controls	60	58	Ref	Ref

*P value ≤0.05

For all LHC cases, eating during application was a very common habit when compared to controls (OR 2.16, 95% CI 1.09-4.47). The habit of smoking and eating at the same time during application had a statistically significant association with total LHC cases (OR 18.18, 95% CI 2.38-381.17).

Using logistic regression analysis, pesticide exposure was found to be independently associated with all cases of LHC (OR 1.41, 95% CI 1.00-2.00) and leukaemia (OR 2.05, 95% CI 1.02-4.12) after controlling for the following confounders: age, sex, smoking and first-degree family history of all types of cancer, family history of LHC and family history of immunological disorders. The results remained almost the same for polytomous regression analysis (Table 7).

**Table 7:** Pesticide exposure adjusted for age, smoking and family history by the use of two different statistical methods.

OR (95%CI)	LHC Cases	MPD	MDS	Leukaemia	Lymphoma	PcD
Logistic Regression	1.41* (1.00-2.00)	1.07 (0.57-1.98)	1.44 (0.74-2.79)	2.05* (1.02-4.12)	1.43 (0.77-2.64)	1.48 (0.70-3.12)
Polytomous Regression	1.42* (1.00-2.00)	1.05 (0.57-1.93)	1.51 (0.78-2.89)	2.04* (1.02-4.08)	1.42 (0.77-2.61)	1.45 (0.67-3.03)

*P value ≤0.05

## Discussion

Our study found statistically significant associations between exposure to pesticides and all cases of LHC and leukaemia, after controlling for confounding factors; furthermore, a dose-response effect was also seen for LHC cases, MDS and leukaemia. Although it was not possible to estimate the risk of the specific occupations, due to the low number of participants, it was determined that agricultural occupations were associated with MDS and leukaemia. These results are consistent with previous studies of farmers, which revealed an elevated risk for LHC [3-7]. It is known that farmers apply agricultural chemicals, including pesticides and many of them are known or suspected human carcinogens [24-27].

The study explored the impact of the type of pesticide application on the development of LHC. Spraying was found to be negatively associated with MPD and MDS, whereas seed treatment was found to be associated with MDS. The number of cases and controls who managed to recall in detail the pesticide products used and the type of application for each one was small compared to the number of participants who in general declared exposure to pesticides. This could be explained by the fact that most of them made use of pesticides many years earlier. Moreover, many subjects were illiterate, especially the women, and the brand names of many products were in English, which made them much difficult to recall. During seed treatment, chemical or biological substances are applied to seeds or vegetative propagation materials to control disease organisms, insects or other pests. Today, most seeds (including corn, wheat and cotton) that have a seed coat surrounding an embryo are treated with pesticide before planting. In Greece this type of formulation was introduced over the last 15-20 years. This is particularly important for the study given that the majority of the cultivated areas of Thessaly are corn and cotton farms. Usually, for cotton farms, the number of applications per year is not high, but the quantities of plant protection products used are larger than among other types of crops. Farmers may be exposed during transportation and through manual equipment filling which is a practice associated with high exposure. In addition, farmers are often unaware that seeds are treated with pesticides or, even if they are aware, the pesticides are not visible, thus farmers usually underestimate the chemical exposure. In comparing the various types of pesticide application, it is presumed that during spraying most protective measures were adopted. The handling of treated seeds without gloves in combination with the results regarding eating during pesticide application could give a further explanation of the study findings. Another hypothesis is that chemicals used during spraying are less toxic than those used in the other types of application. We are now conducting a further study to investigate the effects on pesticide appliers of the type of exposure (spraying or treated seeds) using biomonitoring (metabolites, DNA damage and enzymatic polymorphisms in urine and blood samples).

Pesticide exposure is complicated because many pesticides are mutagenic, teratogenic or carcinogenic whereas others are not, and their formulations are complex and confidential proprietary information. In addition, there are many possible routes of exposure including ingestion, dermal, inhalation and ocular. Pesticides have diverse chemical and biological modes of action and, in addition, their formulations may contain emulsifiers, carriers and dispersants, as well as a variety of agents that are used to formulate liquids, powders and mists. In this respect, it must be noted that extrapolation and generalization of the results is difficult due to the different pesticide formulations used and the complex combinations applied, depending on the region, crop, season, etc. Furthermore, farmers generally mix different pesticide chemicals and information on the particular adverse effects of a defined compound is not enough to adequately evaluate the real genotoxic risk related to complex mixtures [28]. Due to the low numbers of exposed subjects in some subtype categories, definite conclusions could not be drawn for specific chemicals.

Pesticides are typically purchased in a concentrated form and must be diluted prior to application. The diluted material is then transferred to application containers. This mixing and loading process provides opportunities for greater pesticide exposure due to contact with contaminated surfaces, splashes and spills. Pesticides used in recent years are chemically different from those used in the past and are handled under better hygienic conditions.

One important result from this study is that smoking and eating during pesticide application was associated with the total LHC cases. In all the relevant associations found, the odds ratios were quite high and suggested a possible association between smoking and eating during pesticide application and the diseases under investigation (OR from 2 to 10). Moreover, when eating and smoking were reportedly carried out simultaneously during pesticide application an even higher risk was documented (OR 18) for all LCH. Previous studies demonstrated various results regarding smoking habit and farming or pesticide exposure. Smoking was found to be a weak risk factor for developing leukaemia but it was more important for women [29,30]. Regarding the development of multiple myeloma, farming was found to be a risk factor, but smoking habit was not found to be statistically significant factor [31]. In a cohort of individuals exposed to pesticides, mortality from leukaemia and smoking related diseases were high [32]. To our knowledge there has been no research investigating smoking during pesticide application and its association with LHC. It is possible that smoking and eating during pesticide application may increase exposure to pesticides via ingestion and/or dermal absorption. Another interesting hypothesis is that simultaneous smoking and pesticide application may produce a synergistic effect.

The present study has several limitations. There may be a selection bias since the study was hospital-based. In order to control this source of bias, diseases known to be related to agricultural exposure, were excluded during the selection of controls. In addition, there was no incentive for participation and the participants were not aware of the aim of the study. Another possible limitation of the study is related to the collection of prevalent cases. A study based on the collection of incident cases would have possibly provided more valid results, although it must be noted that there is no cancer registry in Greece and this is an obstacle for epidemiological studies. Cases with a relatively recent date of diagnosis were enrolled and we made the assumption that patients with these kinds of diseases usually enter the hospital when recently diagnosed for the first follow-up and treatment. It was very unlikely that cases with a very short or a very long survival period or in complete remission would be identified. Moreover, the diseases included in the study are not very common in Greece. However, we assume that the process of selecting cases did not influence our results substantially.

Cases could be considered representative of the total population of patients with LHC in the region, whereas controls could not be considered representative of the healthy population since they were chosen from the same hospitals as cases. In addition, controls could also be considered as matched to cases with regards to residence and other environmental factors, since the hospitals have a given catchment area. It is possible that misclassification of disease status may have occurred for the control group. Cases were histologically confirmed but controls were not examined histologically for the absence of LHC and were admitted as declared. However, the probability that the control group included subjects with LHC is very low because of the low incidence of these cases in the general population. The different response rates of cases and controls can be explained by the physical and emotional states of the cases. Some of them were undergoing chemotherapy; others were in the acute or the final phase of the disease. With respect to the various conditions, participation was not insisted upon and, according to the protocol of the study, help from a familiar person or hospital assistant was not allowed. Although there was no laboratory confirmation of exposure status, the exposure assessment in this study could be considered an improvement over other similar research studies. Whereas previous studies on pesticide exposure were limited to qualitative exposure measurements, this study attempted to quantify cumulative lifetime exposure by incorporating measures of duration and intensity of exposure to specific pesticides. This approach made it possible to reveal a dose-response relationship between pesticide exposure and LHC. Observational bias could have occurred since the interviewer was not blinded and the status of the participant (case or control) was known to the interviewer. Recall bias could also have occurred, especially among LHC cases. In comparison to controls, they may have made an extra effort to recall possible risk factors for their disease and described more intense exposure. On the other hand, controls may have paid less attention to the questions of the researcher and the possible exposure risks. To minimize this problem for both cases and controls, a list of the most common commercial names of pesticides was provided during the interview, including the target pesticide classification. Concerning protective measures, there is no certainty that subjects who reported use of PPE used the appropriate measures for each phase of the procedure. Cases could be more likely to declare no use of PPE.

Smoking per se is an independent risk factor for developing leukaemia and, for this reason, it was included in the logistic regression model as a confounder. However, the results would have been better if we had obtained data on pack-years for smoking. The small number of participants explains the very broad 95% CI for smoking habit during pesticide application.

## Conclusions

The causality of LHC is multifactorial. In this study, LHC and especially leukaemia were associated with pesticide exposure after controlling for confounders. Moreover, a dose-response effect was observed. Smoking and eating during pesticide application were identified as factors that were associated with increased risk for LHC. Further studies and confirmatory results are needed in order to establish a causal association between exposure to pesticides and LHC.

Several poor work practices for handling pesticides were reported by the study participants. Training and educational campaigns focusing on appropriate use of pesticides should be encouraged.

## Abbreviations

**CI**: Confidence interval; **HD**: Hodgkin's disease; **LHC**: lymphoheamatopoietic cancers; **MDS**: myelodysplastic syndrome; **MPD**: myeloproliferative disorder; **NHL**: non Hodgkin lymphoma; **OR**: odds ratio; **PcD**: plasmacell disease; **PPE**: personal protective equipment; **SD**: standard deviation

## Competing interests

The authors declare that they have no competing interests.

## Authors' contributions

CH is guarantor. CH and MK had a role in conception and design. MK and NP collected participants. MK performed the interviews. NB conducted the statistical analysis. CH, MK, GR and GMH interpreted data. All authors took part in drafting the article or revising it for critically important intellectual content and all gave final approval of the version to be published.

## Pre-publication history

The pre-publication history for this paper can be accessed here:

http://www.biomedcentral.com/1471-2458/11/5/prepub

## Supplementary Material

Additional file 1 **Case control study questionnaire on lymphohaematopoietic cancers**. The structured questionnaire used for both cases and controls. It includes the following sections: 1) demographics, 2) residence, 3) occupation, 4) exposure and agricultural practices, 5) family history and 6) habits and medical history. Click here for file
