# Supplementary material for: Pesticide exposure and lymphohaematopoietic cancers: a case-control study in an agricultural region (Larissa, Thessaly, Greece)
Source: BMC Public Health. 2011 Jan 4;11:5. doi: 10.1186/1471-2458-11-5 (PMC3022699; doi:10.1186/1471-2458-11-5)
Supplement: Additional file 1 — Case control study questionnaire on lymphohaematopoietic cancers. The structured questionnaire used for both cases and controls. It includes the following sections: 1) demographics, 2) residence, 3) occupation, 4) exposure and agricultural practices, 5) family history and 6) habits and medical history. [file 1471-2458-11-5-S1.DOCX]

| Case-control study questionnaire on Lymphohematopoietic cancers |
| --- |

| **1** | | **STATUS** | | | | | | | | | Case 🞎 Control 🞎 | | | | | | | | | | | | | |
| --- | --- | --- | --- | --- | --- | --- | --- | --- | --- | --- | --- | --- | --- | --- | --- | --- | --- | --- | --- | --- | --- | --- | --- | --- |
|  | | | | | | | | | | |  | | | | | | | | | | | | | |
| **2** **PERSONAL DATA** | | | | | | | | | | |  | | | | | | | | | | | | | |
| **2.1** | | | | Surname: | | | | | | |  | | | | | | | | | | | | | |
| **2.2** | | | | Name: | | | | | | |  | | | | | | | | | | | | | |
| **2.3** | | | | Name of father: | | | | | | |  | | | | | | | | | | | | | |
|  | | | | | | | | | | | | | | | | | | | | | | | | |
| **3** | | Sex: | | | | | | | | | Male 🞎 Female 🞎 | | | | | | | | | | | | | |
|  | | | | | | | | | | | | | | | | | | | | | | | | |
| **4** | | Age: | | | | | | | | |  | | | | | | | | | | | | | |
|  | | | | | | | | | | | | | | | | | | | | | | | | |
| **5** | | Occupation: | | | | | | | | | | | | | | | | | | | | | | |
|  | | | | Farmer: | | | | 🞎 | | | | | | | | | | | | | | | | |
|  | | | | Pesticide applicator: | | | | 🞎 | | | | | | | | | | | | | | | | |
|  | | | | Seasonal farm-worker: | | | | 🞎 | | | | | | | | | | | | | | | | |
|  | | | | Animal breeder: | | | | 🞎 | | | | | | | | | | | | | | | | |
|  | | | | Unemployed: | | | | 🞎 | | | | | | | | | | | | | | | | |
|  | | | | Other: | | | | 🞎 | | (define): | | | | | | | | | | | | | | |
|  | | | | | | | | | | | | | | | | | | | | | | | | |
| **6** Histological type (case) / Department (control): | | | | | | | | | | | | | | | | | |  | | | | | | |
|  | | | | | | | | | | | | | | | | | | | | | | | | |
| **7** Date of Diagnosis (case) / Interview (control): | | | | | | | | | | | | | | | | | |  | | | | | | |
|  | | | | | | | | | | | | | | | | | | | | | | | | |
| **8** Hospital: | | | | | | | | | | | | | | | | | |  | | | | | | |
|  | | | | | | | | | | | | | | | | | | | | | | | | |
| **9** Code number: | | | | | | | | | | | | | | | | | |  | | | | | | |
|  | | | | | | | | | | | | | | | | | | | | | | | | |
| **10** | | **RESIDENCE (chronologically)** | | | | | | | | | | | | | | | | | | | | | | |
|  | | | | | | | | | | | | | | | | | | | | | | | | |
| **10.1** | | | | | Urban 🞎 Suburban 🞎 Rural 🞎 | | | | | | | | | | | | | | | | | | | |
| **10.1.1** | | | | | Region: | | | | | | | | | **10.1.2** Code of region: | | | | | | | | | | |
| **10.1.3** | | | | | City/Village: | | | | | | | | | **10.1.4** Code: | | | | | | | | | | |
| **10.1.5** | | | | | Address: | | | | | | | | | **10.1.6** Number: | | | | | | | | | | |
| **10.1.7** | | | | | Years of residence: | | | | | | | | |  | | | | | | | | | | |
|  | | | | | | | | | | | | | | | | | | | | | | | | |
| **10.2** | | | | | Urban 🞎 Suburban 🞎 Rural 🞎 | | | | | | | | | | | | | | | | | | | |
| **10.2.1** | | | | | Region: | | | | | | | | | **10.2.2** Code of region: | | | | | | | | | | |
| **10.2.3** | | | | | City/Village: | | | | | | | | | **10.2.4** Code: | | | | | | | | | | |
| **10.2.5** | | | | | Address: | | | | | | | | | **10.2.6** Number: | | | | | | | | | | |
| **10.2.7** | | | | | Years of residence: | | | | | | | | |  | | | | | | | | | | |
|  | | | | | | | | | | | | | | | | | | | | | | | | |
| **10.3** | | | | | Urban 🞎 Suburban 🞎 Rural 🞎 | | | | | | | | | | | | | | | | | | | |
| **10.3.1** | | | | | Region: | | | | | | | | | **10.3.2** Code of region: | | | | | | | | | | |
| **10.3.3** | | | | | City/Village: | | | | | | | | | **10.3.4** Code: | | | | | | | | | | |
| **10.3.5** | | | | | Address: | | | | | | | | | **10.3.6** Number: | | | | | | | | | | |
| **10.3.7** | | | | | Years of residence: | | | | | | | | |  | | | | | | | | | | |
|  | | | | | | | | | | | | | | | | | | | | | | | | |
| **11** | | **EDUCATION** | | | | | | | | | | | | | | | | | | | | | | |
|  | | | | None: | | | | 🞎 | | | | | | | | | | | | | | | | |
|  | | | | Elementary: | | | | 🞎 | | | | | | | | | | | | | | | | |
|  | | | | Intermediate: | | | | 🞎 | | | | | | | | | | | | | | | | |
|  | | | | Lyceum: | | | | 🞎 | | | | | | | | | | | | | | | | |
|  | | | | College: | | | | 🞎 | | | | | | | | | | | | | | | | |
|  | | | | University: | | | | 🞎 | | | | | | | | | | | | | | | | |
|  | | | | | | | | | | | | | | | | | | | | | | | | |
| **12** | | **INSURANCE** | | | | | | | | | | | | | | | | | | | | | | |
| **11.1** | | | | Farmer’s (OGA): | | | | 🞎 | | | | | | | | | | | | | | | | |
| **11.2** | | | | Public Insurance Institute (IKA): | | | | 🞎 | | | | | | | | | | | | | | | | |
| **11.3** | | | | State Industrial and Commercial Fund (TEBE): | | | | 🞎 | | | | | | | | | | | | | | | | |
| **11.4** | | | | Personal: | | | | 🞎 | | | | | | | | | | | | | | | | |
| **11.5** | | | | None: | | | | 🞎 | | | | | | | | | | | | | | | | |
| **11.6** | | | | Other: | | | | 🞎 | | (define): | | | | | | | | | | | | | | |
|  | | | |  | | | |  | | | | | | | | | | | | | | | | |
|  | | | | | | | | | | | | | | | | | | | | | | | | |
| **13** | | **MAIN OCCUPATION (chronologically)** | | | | | | | | | | | | | | | | | | | | | | |
|  | | | | | | | | | | | | | | | | | | | | | | | | |
| **13.1.1** | | | | | Occupation: | | | | | | | | | | | | **13.1.2** | | | | Duration in years: | | | |
| **13.1.3** | | | | | Urban 🞎 Suburban 🞎 Rural 🞎 | | | | | | | | | | | | | | | | | | | |
| **13.1.4** | | | | | Region: | | | | | | | | | **13.1.5** City/Village: | | | | | | | | | | |
|  | | | | | | | | | | | | | | | | | | | | | | | | |
| **13.2.1** | | | | | Occupation: | | | | | | | | | | | | **13.2.2** | | | | Duration in years: | | | |
| **13.2.3** | | | | | Urban 🞎 Suburban 🞎 Rural 🞎 | | | | | | | | | | | | | | | | | | | |
| **13.2.4** | | | | | Region: | | | | | | | | | **13.2.5** City/Village: | | | | | | | | | | |
|  | | | | |  | | | | | | | | |  | | | | | | | | | | |
| **13.3.1** | | | | | Occupation: | | | | | | | | | | | | **13.3.2** | | | | Duration in years: | | | |
| **13.3.3** | | | | | Urban 🞎 Suburban 🞎 Rural 🞎 | | | | | | | | | | | | | | | | | | | |
| **13.3.4** | | | | | Region: | | | | | | | | | **13.3.5** City/Village: | | | | | | | | | | |
|  | | | | |  | | | | | | | | |  | | | | | | | | | | |
| **13.4.1** | | | | | Occupation: | | | | | | | | | | | | **13.4.2** | | | | Duration in years: | | | |
| **13.4.3** | | | | | Urban 🞎 Suburban 🞎 Rural 🞎 | | | | | | | | | | | | | | | | | | | |
| **13.4.4** | | | | | Region: | | | | | | | | | **13.4.5** City/Village: | | | | | | | | | | |
|  | | | | |  | | | | | | | | |  | | | | | | | | | | |
| **13.5.1** | | | | | Occupation: | | | | | | | | | | | | **13.5.2** | | | | Duration in years: | | | |
| **13.5.3** | | | | | Urban 🞎 Suburban 🞎 Rural 🞎 | | | | | | | | | | | | | | | | | | | |
| **13.5.4** | | | | | Region: | | | | | | | | | **13.5.5** City/Village: | | | | | | | | | | |
|  | | | | |  | | | | | | | | |  | | | | | | | | | | |
| **14** | | **OTHER ACTIVITIES AND HOBBIES** | | | | | | | | | | | | | | | | | | | | | | |
|  | | | | | | | | | | | | | | | | | | | | | | | | |
| **14.1.1** | | | | | Type: | | | | | | | | | | | | **14.1.2** | | | | Duration in years: | | | |
| **14.1.3** | | | | | Urban 🞎 Suburban 🞎 Rural 🞎 | | | | | | | | | | | | | | | | | | | |
| **14.1.4** | | | | | Region: | | | | | | | | | **14.1.5** City/Village: | | | | | | | | | | |
|  | | | | | | | | | | | | | | | | | | | | | | | | |
|  | | | | | | | | | | | | | | | | | | | | | | | | |
| **14.2.1** | | | | | Type: | | | | | | | | | | | | **14.2.2** | | | | Duration in years: | | | |
| **14.2.3** | | | | | Urban 🞎 Suburban 🞎 Rural 🞎 | | | | | | | | | | | | | | | | | | | |
| **14.2.4** | | | | | Region: | | | | | | | | | **14.2.5** City/Village: | | | | | | | | | | |
|  | | | | | | | | | | | | | | | | | | | | | | | | |
| **14.3.1** | | | | | Type: | | | | | | | | | | | | **14.3.2** | | | | Duration in years: | | | |
| **14.3.3** | | | | | Urban 🞎 Suburban 🞎 Rural 🞎 | | | | | | | | | | | | | | | | | | | |
| **14.3.4** | | | | | Region: | | | | | | | | | **14.3.5** City/Village: | | | | | | | | | | |
|  | | | | | | | | | | | | | | | | | | | | | | | | |
| **15** | | **TYPE OF CROPS** | | | | | | | | | | | | | | | | | | | | | | |
|  | | | | | | | | | | | | | | | | | | | | | | | | |
| **15.1.1** | | | | | Cereal crops (wheat, barley, oats, rye etc): | | | | | | | | | | | | | | | | | | 🞎 | |
| **15.1.2** | | | | | Duration: | | | | | | | | | | | | | | | | | | (years) | |
| **15.1.3** | | | | | Area: | | | | | | | | | | | | | | | | | | (Km^2^) | |
|  | | | | | | | | | | | | | | | | | | | | | | | | |
| **15.2.1** | | | | | Combinable crops (cotton, tobacco, corn, beetroot, potatoes): | | | | | | | | | | | | | | | | | | 🞎 | |
| **15.2.2** | | | | | Duration: | | | | | | | | | | | | | | | | | | (years) | |
| **15.2.3** | | | | | Area: | | | | | | | | | | | | | | | | | | (Km^2^) | |
|  | | | | | | | | | | | | | | | | | | | | | | | | |
| **15.3.1** | | | | | Tree fruit (citrus fruit, apple etc): | | | | | | | | | | | | | | | | | | 🞎 | |
| **15.3.2** | | | | | Duration: | | | | | | | | | | | | | | | | | | (years) | |
| **15.3.3** | | | | | Area: | | | | | | | | | | | | | | | | | | (Km^2^) | |
|  | | | | | | | | | | | | | | | | | | | | | | | | |
| **15.4.1** | | | | | Butterfly-blossoms (clover, lentil, bean, mange-tout, vetch etc): | | | | | | | | | | | | | | | | | | 🞎 | |
| **15.4.2** | | | | | Duration: | | | | | | | | | | | | | | | | | | (years) | |
| **15.4.3** | | | | | Area: | | | | | | | | | | | | | | | | | | (Km^2^) | |
|  | | | | | | | | | | | | | | | | | | | | | | | | |
| **15.5.1** | | | | | Decorative plants: | | | | | | | | | | | | | | | | | | 🞎 | |
| **15.5.2** | | | | | Duration: | | | | | | | | | | | | | | | | | | (years) | |
| **15.5.3** | | | | | Area: | | | | | | | | | | | | | | | | | | (Km^2^) | |
|  | | | | | | | | | | | | | | | | | | | | | | | | |
| **15.6.1** | | | | | Other (define): | | | | | | | | | | | | | | | | | | 🞎 | |
| **15.6.2** | | | | | Duration: | | | | | | | | | | | | | | | | | | (years) | |
| **15.6.3** | | | | | Area: | | | | | | | | | | | | | | | | | | (Km^2^) | |
|  | | | | | | | | | | | | | | | | | | | | | | | | |
| **16** | | **ADJACENT FARMS** | | | | | | | | | | | | | | | | | | | | | | |
|  | | | | | | | | | | | | | | | | | | | | | | | | |
| **16.1.1** | | | | | Cereal crops (wheat, barley, oats, rye etc): | | | | | | | | | | | | | | | | | | 🞎 | |
| **16.1.2** | | | | | Duration: | | | | | | | | | | | | | | | | | | (years) | |
| **16.1.3** | | | | | Area: | | | | | | | | | | | | | | | | | | (Km^2^) | |
|  | | | | | | | | | | | | | | | | | | | | | | | | |
| **16.2.1** | | | | | Combinable crops (cotton, tobacco, corn, beetroot, potatoes): | | | | | | | | | | | | | | | | | | 🞎 | |
| **16.2.2** | | | | | Duration: | | | | | | | | | | | | | | | | | | (years) | |
| **16.2.3** | | | | | Area: | | | | | | | | | | | | | | | | | | (Km^2^) | |
|  | | | | | | | | | | | | | | | | | | | | | | | | |
| **16.3.1** | | | | | Tree fruit (citrus fruit, apple etc): | | | | | | | | | | | | | | | | | | 🞎 | |
| **16.3.2** | | | | | Duration: | | | | | | | | | | | | | | | | | | (years) | |
| **16.3.3** | | | | | Area: | | | | | | | | | | | | | | | | | | (Km^2^) | |
|  | | | | | | | | | | | | | | | | | | | | | | | | |
| **16.4.1** | | | | | Butterfly-blossoms (clover, lentil, bean, mange-tout, vetch etc): | | | | | | | | | | | | | | | | | | 🞎 | |
| **16.4.2** | | | | | Duration: | | | | | | | | | | | | | | | | | | (years) | |
| **16.4.3** | | | | | Area: | | | | | | | | | | | | | | | | | | (Km^2^) | |
|  | | | | | | | | | | | | | | | | | | | | | | | | |
| **16.5.1** | | | | | Decorative plants: | | | | | | | | | | | | | | | | | | 🞎 | |
| **16.5.2** | | | | | Duration: | | | | | | | | | | | | | | | | | | (years) | |
| **16.5.3** | | | | | Area: | | | | | | | | | | | | | | | | | | (Km^2^) | |
|  | | | | | | | | | | | | | | | | | | | | | | | | |
| **16.6.1** | | | | | Other (define): | | | | | | | | | | | | | | | | | | 🞎 | |
| **16.6.2** | | | | | Duration: | | | | | | | | | | | | | | | | | | (years) | |
| **16.6.3** | | | | | Area: | | | | | | | | | | | | | | | | | | (Km^2^) | |
|  | | | | | | | | | | | | | | | | | | | | | | | | |
| **17** | | **Common pests** | | | | | | | | | | | | | | | | | | | | | | |
|  | | | | Insects: | | | | 🞎 | | | | | | | | | | | | | | | | |
|  | | | | Acari: | | | | 🞎 | | | | | | | | | | | | | | | | |
|  | | | | Nematodes: | | | | 🞎 | | | | | | | | | | | | | | | | |
|  | | | | Fungi: | | | | 🞎 | | | | | | | | | | | | | | | | |
|  | | | | Weeds: | | | | 🞎 | | | | | | | | | | | | | | | | |
|  | | | | | | | | | | | | | | | | | | | | | | | | |
| **18** | | **Do you apply the pesticides by yourself?** | | | | | | | | | | Yes 🞎 No 🞎 | | | | | | | | | | | | |
|  | | | | | | | | | | | | | | | | | | | | | | | | |
| **19** | | **PLANT PROTECTIVE PRODUCTS** | | | | | | | | | | | | | | | | | | | | | | |
|  | | | | | | | | | | | | | | | | | | | | | | | | |
|  | Name | | | | | Applications/year | | | | | | | | | Years | | | | | | | Region | Type of application | |
|  | 1. | | | | |  | | | | | | | | |  | | | | | | |  | (a) (b) (c) | |
|  | 2. | | | | |  | | | | | | | | |  | | | | | | |  | (a) (b) (c) | |
|  | 3. | | | | |  | | | | | | | | |  | | | | | | |  | (a) (b) (c) | |
|  | 4. | | | | |  | | | | | | | | |  | | | | | | |  | (a) (b) (c) | |
|  | 5. | | | | |  | | | | | | | | |  | | | | | | |  | (a) (b) (c) | |
|  | 6. | | | | |  | | | | | | | | |  | | | | | | |  | (a) (b) (c) | |
|  | 7. | | | | |  | | | | | | | | |  | | | | | | |  | (a) (b) (c) | |
|  | | | | | | | | | | | | | | | | | | | | | | | | |
| (a) spraying (b) during planting (c) seed treatment | | | | | | | | | | | | | | | | | | | | | | | | |
| Active principle (to be completed by the research team) | | | | | | | | | | | | | | | | | | | | | | | | |
|  | 1. | | | | | | | | | | | | | | | | | | | | | | | |
|  | 2. | | | | | | | | | | | | | | | | | | | | | | | |
|  | 3. | | | | | | | | | | | | | | | | | | | | | | | |
|  | 4. | | | | | | | | | | | | | | | | | | | | | | | |
|  | 5. | | | | | | | | | | | | | | | | | | | | | | | |
|  | 6. | | | | | | | | | | | | | | | | | | | | | | | |
|  | 7. | | | | | | | | | | | | | | | | | | | | | | | |
|  | 8. | | | | | | | | | | | | | | | | | | | | | | | |
|  | | | | | | | | | | | | | | | | | | | | | | | | |
| **20** Do you adhere to the guidelines and precautions of the labels of the pesticides? | | | | | | | | | | | | | | | | | | | | Always 🞎 Sometimes 🞎 Never 🞎 | | | | |
|  | | | | |  | | | | | | | | | | | | | | | | | |  | |
| **21** Are you familiar with the caution signs of the products? | | | | | | | | | | | | | | | | | | | | Yes 🞎 Not all 🞎 No 🞎 | | | | |
|  | | | | |  | | | | | | | | | | | | | | | | | |  | |
| **22** Are you familiar with the first aid help after accidental poisoning? | | | | | | | | | | | | | | | | | | | | Yes 🞎 Not always 🞎 No 🞎 | | | | |
|  | | | | | | | | | | | | | | | | | | | | | | | | |
| **23** Is the first aid station easily reachable? | | | | | | | | | | | | | | | | | | | | Yes 🞎 Not always 🞎 No 🞎 | | | | |
|  | | | | | | | | | | | | | | | | | | | | | | | | |
|  | | | | | | | | |  | | | | | | | | | | | | | | | |
| **24** Personal Protective Equipment (PPE) | | | | | | | | | Gloves 🞎 Mask 🞎 Uniform 🞎 Boots 🞎 Other 🞎 | | | | | | | | | | | | | | | |
|  | | | | | | | | | (define) | | | | | | | | | | | | | | | |
|  | | | | | | | | | | | | | | | | | | | | | | | | |
| **25** Use of PPE during preparation (transportation, mixing etc) | | | | | | | | | | | | | | | | | | | | Always 🞎 Sometimes 🞎 Never 🞎 | | | | |
|  | | | | | | | | | | | | | | | | | | | | | | | | |
| **26** Use of PPE during application | | | | | | | | | | | | | | | | | | | | Always 🞎 Sometimes 🞎 Never 🞎 | | | | |
|  | | | | | | | | | | | | | | | | | | | | | | | | |
| **27** Use of PPE during first entrances after application | | | | | | | | | | | | | | | | | | | | Always 🞎 Sometimes 🞎 Never 🞎 | | | | |
|  | | | | | | | | | | | | | | | | | | | | | | | | |
| **28** Use of PPE during cleaning of machinery, clothes etc | | | | | | | | | | | | | | | | | | | | Always 🞎 Sometimes 🞎 Never 🞎 | | | | |
|  | | | | | | | | | | | | | | | | | | | | | | | | |
| **30** | | Use of machinery | | | | | | | | | | Yes 🞎 No 🞎 | | | | | | | | | | | | |
| Define | | | | | | | | | | | | | | | | | | | | | | | | |
|  | | | | | | | | | | | | | | | | | | | | | | | | |
| **31** Do you change clothes before entering home? | | | | | | | | | | | | | | | | | | | | Always 🞎 Sometimes 🞎 Never 🞎 | | | | |
|  | | | | | | | | | | | | | | | | | | | | | | | | |
| **32** Do you smoke during farming? | | | | | | | | | | | | | | | | | | | | Always 🞎 Sometimes 🞎 Never 🞎 | | | | |
|  | | | | | | | | | | | | | | | | | | | | | | | | |
| **33** Do you eat or drink during farming? | | | | | | | | | | | | | | | | | | | | Always 🞎 Sometimes 🞎 Never 🞎 | | | | |
|  | | | | | | | | | | | | | | | | | | | | | | | | |
| **34** Does accidental poisoning ever occurred to you? | | | | | | | | | | | | |  | | | | | | | | | | | |
|  | | | | | | | | | | | | | | | | | | | | | | | | |
| **35** Do you consult a specialist (e.g. phytopathologist)? | | | | | | | | | | | | | | | | | | | | Always 🞎 Sometimes 🞎 Never 🞎 | | | | |
|  | | | | | | | | | | | | | | | | | | | | | | | | |
| **36** Do you usually buy excessive product quantity than used? | | | | | | | | | | | | | | | | | | | | Always 🞎 Sometimes 🞎 Never 🞎 | | | | |
|  | | | | | | | | | | | | | | | | | | | | | | | | |
| **37** | | How do you manage the excessive quantity? | | | | | | | | | | | | |  | | | | | | | | | |
| Define | | | | | | | | | | | | | | | | | | | | | | | | |
|  | | | | | | | | | | | | | | | | | | | | | | | | |
| **38** | | Is there a special destination for pesticide eluants? | | | | | | | | | | | | | | | | | Yes 🞎 No 🞎 | | | | | |
|  | | | | | | | | | | | | | | | | | | | | | | | | |
| **39** | | Do you have a special place of storage? | | | | | | | | | | | | | | | | | Yes 🞎 No 🞎 | | | | | |
|  | | | | | | | | | | | | | | | | | | | | | | | | |
| **40** | | The storage place is equipped with | | | | | | | | | | | | | | | | | | | | | | |
|  | | | | Emergency exit: | | | | 🞎 | | | | | | | | | | | | | | | | |
|  | | | | Ventilation: | | | | 🞎 | | | | | | | | | | | | | | | | |
|  | | | | Proper signs: | | | | 🞎 | | | | | | | | | | | | | | | | |
|  | | | | Chemical detector: | | | | 🞎 | | | | | | | | | | | | | | | | |
|  | | | | | | | | | | | | | | | | | | | | | | | | |
| **41** | | Are your crops under an integrated farm system? | | | | | | | | | | | | | | Yes 🞎 No 🞎 Define: | | | | | | | | |
|  | | | | | | | | | | | | | | | | | | | | | | | | |
| **42** | | **ANIMALS** | | | | | | | | | | Yes 🞎 No 🞎 | | | | | | | | | | | | |
|  | | | | | | | | | | | | | | | | | | | | | | | | |
|  | | | | | | | | | | | | | | | | | | | | | | | | |
|  | | | | | | | | | | | | | | | | | | | | | | | | |
|  | | | | | | | | | | | | | | | | | | | | | | | | |
|  | | | | | | | | | | | | | | | | | | | | | | | | |
| **43** | | Industrial 🞎 | | | | | | | | | | | | | | | | | | | | | | |
|  | | | | Cattle: | | | | 🞎 | | | | | | | | | | | | | | | | |
|  | | | | Sheep: | | | | 🞎 | | | | | | | | | | | | | | | | |
|  | | | | Horse: | | | | 🞎 | | | | | | | | | | | | | | | | |
|  | | | | Swine: | | | | 🞎 | | | | | | | | | | | | | | | | |
|  | | | | Poultry: | | | | 🞎 | | | | | | | | | | | | | | | | |
|  | | | | | | | | | | | | | | | | | | | | | | | | |
| **44** | | Companion animals 🞎 | | | | | | | | | | | | | | | | | | | | | | |
|  | | | | Cat: | | | | 🞎 | | | | | | | | | | | | | | | | |
|  | | | | Dog: | | | | 🞎 | | | | | | | | | | | | | | | | |
|  | | | | Other: | | | | 🞎 | | (define) | | | | | | | | | | | | | | |
|  | | | | | | | | | | | | | | | | | | | | | | | | |
| **45** | | **HABITS** | | | | | | | | | | | | | | | | | | | | | | |
|  | | Smoking | | | | | | | | | | | | | | | | | | | | | | |
|  | | | | Never: | | | | | | | | | | | | | | 🞎 | | | | | | |
|  | | | | Occasionally (define number/week): | | | | | | | | | | | | | | 🞎 | | | | | | |
|  | | | | Current (define number/day): | | | | | | | | | | | | | | 🞎 | | | | | | |
|  | | | | Former smoker (define data of cessation): | | | | | | | | | | | | | | 🞎 | | | | | | |
|  | | | | | | | | | | | | | | | | | | | | | | | | |
| **46** | | Alcohol (1 drink = 1 bottle of beer or a glass of wine or a dose of liqueur or a dose of cocktail) | | | | | | | | | | | | | | | | | | | | | | |
|  | | | | Less than 12 drinks in the whole life: | | | | | | | | | | | | | | 🞎 | | | | | | |
|  | | | | Less than 5 drinks in every occasion: | | | | | | | | | | | | | | 🞎 | | | | | | |
|  | | | | More than 5 drinks in every occasion: | | | | | | | | | | | | | | 🞎 | | | | | | |
|  | | | | More than 5 drinks daily: | | | | | | | | | | | | | | 🞎 | | | | | | |
|  | | | | | | | | | | | | | | | | | | | | | | | | |
| **47** | | **MEDICAL HISTORY** | | | | | | | | | | | | | | | | | | | | | | |
|  | | Family history | | | | | | | | | | | | | | | | | | | | | | |
|  | | All types of cancer | | | | | Yes 🞎 No 🞎 Grade of familiarity | | | | | | | | | | | | | | | | | |
|  | | Cancers of the blood | | | | | Yes 🞎 No 🞎 Grade of familiarity | | | | | | | | | | | | | | | | | |
|  | | Immunological diseases | | | | | Yes 🞎 No 🞎 Grade of familiarity | | | | | | | | | | | | | | | | | |
|  | | | | | | | | | | | | | | | | | | | | | | | | |
| **48** | | Personal history | | | | | | | | | | | | | | | | | | | | | | |
|  | | | Other neoplasms: | | | | | 🞎 | | Cardiovascular disorders: | | | | | | | | | | | | | | 🞎 |
|  | | | Respiratory diseases: | | | | | 🞎 | | Immunologic disorders: | | | | | | | | | | | | | | 🞎 |
|  | | | Inflammatory diseases: | | | | | 🞎 | | Endocrinologic disorders: | | | | | | | | | | | | | | 🞎 |
|  | | | Diabetes Mellitus: | | | | | 🞎 | | Metabolic disorders: | | | | | | | | | | | | | | 🞎 |
|  | | | Other (define): | | | | | 🞎 | |  | | | | | | | | | | | | | |  |
|  | | | | | | | | | | | | | | | | | | | | | | | | |
| **49** | | Chronic use of medications | | | | | | No 🞎 Yes 🞎 (define) | | | | | | | | | | | | | | | | |
|  | | | | | | | | | | | | | | | | | | | | | | | | |
